# Seasonal influence on the essential oil chemical composition of *Hyptis crenata* Pohl ex Benth.: a valuable plant from Marajó, Brazil

**DOI:** 10.3389/fchem.2024.1397634

**Published:** 2024-05-28

**Authors:** Maria Nancy Norat de Lima, Paulo Vinicius Lima Santos, Lucas Botelho Jerônimo, Rian Martins Viana, Joyce Kelly da Silva, William N. Setzer, José Guilherme S. Maia, Pablo Luis B. Figueiredo

**Affiliations:** ^1^ Programa de Pós-Graduação em Química, Instituto de Ciências Exatas e Naturais, Universidade Federal do Pará, Belém, Brazil; ^2^ Laboratório de Química dos Produtos Naturais, Universidade do Estado Pará, Belém, Brazil; ^3^ Programa de Pós-Graduação em Ciências Farmacêuticas, Instituto de Ciências da Saúde, Universidade Federal do Pará, Belém, Brazil; ^4^ Programa de Pós-Graduação em Ciências Ambientais, Centro de Ciências Naturais e Tecnologias, Universidade do Estado do Pará, Belém, Brazil; ^5^ Aromatic Plant Research Center, Lehi, UT, United States; ^6^ Laboratório de Bioprospecção e Biologia Experimental, Universidade Federal do Oeste do Pará, Santarém, Brazil

**Keywords:** salva-do-Marajó, volatiles, monoterpenes, medicinal plant, seasonality

## Abstract

**Introduction:** Essential oils (EOs) from the *Hyptis* genus have been reported as bactericides and fungicides. However, the properties of these oils can be affected by climatic factors, as well as the collection period, which promotes changes in the chemical composition of the oil. In this context, this study aimed to evaluate the climatological influences on the chemical composition of the essential oil from the leaves of *Hyptis crenata*.

**Methods:** The leaves were collected in Marajó island (Brazil) monthly for a year. The EOs were obtained by hydrodistillation and analyzed by Gas Chromatography coupled to Mass Spectrometry (GC-MS). Pearson’s correlation was used to evaluate the relationship between climatic parameters, content, and chemical composition of essential oil; multivariate analysis was used to evaluate the interrelationship between samples and their chemical constituents.

**Results and Discussion:** The constituents with the highest contents (>2.0%) in essential oils during the studied period were 1,8-cineole (28.48% ± 4.32%), α-pinene (19.58% ± 2.29%), camphor (11.98% ± 2.54%), β-pinene (9.19% ± 1.47%), limonene (6.12% ± 3.15%), α-terpineol (2.42% ± 0.25%) and borneol (2.34% ± 0.48%). β-Pinene significantly correlated (*p* < 0.05) with precipitation and humidity. According to the chemometric tools, two groups were formed: chemical profile I, marked by 1,8 cineole, α-pinene, β-pinene, borneol, α-terpineol, and limonene, while group II (July) presented a chemical type characterized by camphor. It is understood that the species in question can be a reliable source of biologically active components during different climatic periods in the Amazon. The chemical variability could have significant implications for the pharmaceutical industry and traditional medicine.

## 1 Introduction

*Hyptis crenata* Pohl ex Benth. (Lamiaceae) is an aromatic herb (Campos et al., 2021) that grows spontaneously in sandy and stony soils. In Brazil, this species occurs along the Amazon River, near streams on Marajó Island, Pará, Brazil, where it is popularly known among the local inhabitants as a salva-do-Marajó (Zoghbi et al., 2002).

In the Marajoara region, the *H. crenata* fresh or dried leaves are used to treat liver diseases, stomach pains, and headaches (Rebelo et al., 2009); another constant use is as a flavoring for drinks, scent baths, and incense due to the aromas released by the essential oils contained in the plant (BEZERRA, 2020).

Previous research reported that *Hyptis* species present in their extracts and essential oils bioactive compounds with antibacterial (Jesus et al., 2009; Feitosa-Alcântara et al., 2018) and antifungal (Oliveira et al., 2004), gastroprotective (Diniz et al., 2013), antinociceptive, and anti-inflammatory (de Lima et al., 2023).

Despite the biological effects of *H. crenata* described in the literature, the chemical composition of medicinal species can vary depending on several environmental and physiological factors of the plant, such as growth phase, geographic location, period of the year, climatic season, and solar index, which may change the bioactive properties of natural products such as essential oils (Gobbo-Neto and Lopes, 2007).

According to the main chemical constituents of *H. crenata* essential oil, ten chemical profiles (chemotypes) may occur due to genetic and collection site variation, giving an intraspecific chemical variability (Lima et al., 2023).

Moreover, there is a need to study how climatic parameters alter the chemical composition and consequently the biological activities of essential oils, since these factors appear to be associated with the quality control of natural products such as essential oils (Costa et al., 2022).

*Hyptis crenata* essential oil has a perspective on developing a phytotherapeutic product against pain and inflammation (de Lima et al., 2023). This study aims to evaluate the influence of climatic factors in the Brazilian Amazon on the chemical composition of *H. crenata* essential oil occurring in the Marajó Archipelago.

## 2 Methodology

### 2.1 Plant material and climate data

**FIGURE 1:**
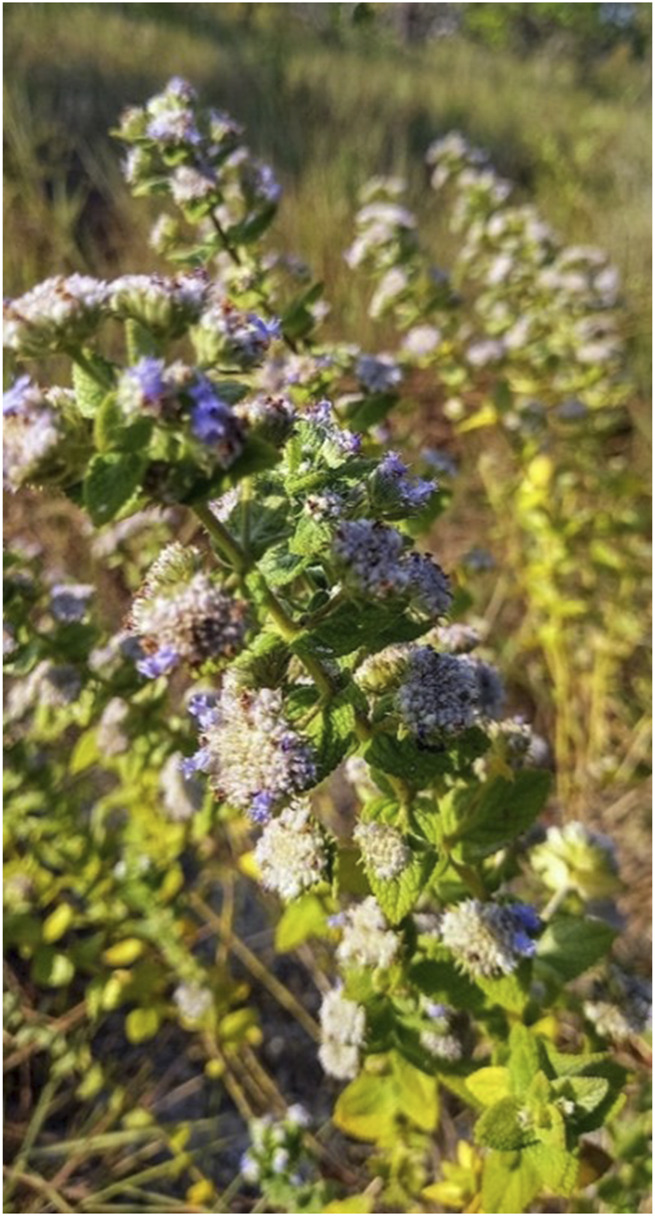
*Hyptis crenata* Pohl ex Benth aerial parts.

*H. crenata* was collected in Vila de Chiquita (rural area, Figure 1) in the city of Salvaterra, Marajó, Pará state, Brazil (Lat. 0°51′43.71″S, Long. 48°37′23.33″W), in accordance with biodiversity protection laws, and the registration of access to genetic heritage under number AEC4B1F (SISGEN).

For the seasonal study, 12 monthly collections were carried out, with approximately 150 g of botanical material, on the first day of each month, starting in September 2021 and ending in August 2022, at 3:00 p.m.

The botanical identification was made by morphological comparison with authentic samples, and a voucher was incorporated into the collection of the Herbario of Museu Paraense Emílio Goeldi in Belém, Pará, under registration number MG-243648.

Climatic data such as atmospheric humidity, rainfall, average temperature, and solar radiation were collected on the website of the National Institute of Meteorology (INMET) of the Brazilian Government. The seasonal parameters were recorded through the automatic station located in Belém, State of Pará, Brazil, which is approximately 78 km as the straight line from the collection site.

### 2.2 Essential oil extraction and yield calculation

The extraction of leaves essential oil from a single specimen of *H. crenata* was carried out using the hydrodistillation technique with a Clevenger-type device over a 3-h period, in triplicate. In this process, 50 g of dried and crushed leaves were added to a 2000-mL glass flask with 100 mL of distilled water. This system was coupled to the condenser, which was cooled at 10°C.

The extracted oils were centrifugated at 3,000 rpm for 5 min to allow total water separation and further dehydrated with anhydrous sodium sulfate (Na_2_SO_4_) under the same conditions. After this dehydration, the masses of the oils were determined using an analytical balance with an accuracy of 0.0001 g, stored in amber glass vials, and kept refrigerated at 5°C (Jerônimo et al., 2024). The essential oil yields were calculated from the moisture-free biomass, relating the plant mass, oil, and residual moisture, according to the Equation below.

$$\%\text{EO}=\left(\frac{\text{obtained}\;\text{oil}\;\text{volume}\;\left(g\right)}{\text{plant}\;\text{material}\;\text{mass}\;\left(g\right)‐\left(\frac{\text{plat}\;\text{material}\;\text{mass}\;\left(g\right)\;x\;\text{humidity}\;\left(\%\right)}{100}\right)}\right)\;x\;100\%$$

The residual moisture content of the leaves was calculated by water loss in an oven at 110°C until the material reached a constant weight.

### 2.3 Analysis of chemical composition

The obtained essential oils were diluted in *n*-hexane in a ratio of 2 μL of oil to 500 μL of solvent and analyzed simultaneously in these two systems: gas chromatography with a flame ionization detector (GC-FID, Shimadzu Corporation, Tokyo, Japan) and gas chromatography with a mass spectrometer (GC-MS, Shimadzu Corporation, Tokyo, Japan) as stabilized protocol (Jerônimo et al., 2024). The system was equipped with an auto-injector: AOC-20i, and an Rtx-5MS silica capillary column (30 m; 0.25 mm; 0.25 μm film thickness) under the following operating conditions: temperature program: 60°C–240°C (3°C/min); injector temperature: 250°C; carrier gas: helium (1 mL/min); injection: split type 1:20 (solution of 5 μL of essential oil: 500 μL of hexane); mass spectra: were obtained by electronic ionization at 70 eV; ion source temperature: 200°C.

To determine the chemical composition, the retention times of each peak (constituents) were converted in retention indices using a homologous series of C_8_–C_40_ *n*-alkanes (Sigma-Aldrich, Milwaukee, WI, United States) according to the linear method of van Den Dool and Kratz (Van Den Dool and Kratz, 1963). Each mass spectrum and retention index were compared with Adams and FFNSC-2 libraries (Adams, 2007; Mondello, 2011). The Relative amounts of individual components were calculated by peak area normalization using the flame ionization detector (GC-FID).

### 2.4 Statistical analysis

Principal component analysis (PCA) was applied to the essential oil components of *H. crenata* leaves (>1.5%) (OriginPro Learning Edition, OriginLab Corporation, Northampton, MA, United States). Hierarchical cluster analysis (HCA) was performed considering the unique distance and Ward linkage. Statistical significance was assessed using the Tukey test (*p* < 0.05) and Pearson correlation coefficients (r) were calculated to determine the relationship between the analyzed climatic parameters (sunlight, relative humidity, temperature, and precipitation), using the GraphPad Prism software, version 8.0.

## 3 Results and discussion

### 3.1 Relationship between essential oil yield and climatic parameters

The climatic parameters: temperature, solar radiation, precipitation, and relative humidity were monitored over the 12 months (September/2021 to August/2022) to evaluate the influence of seasonality on the yield and composition of *H. crenata* leaves essential oil. During the study periods, insolation values ranged from 106.4 h (March) to 253.4 h (August), monthly precipitation from 103.9 mm (August) to 527.4 mm (March), temperature from 25.9°C (January) to 27.6°C (October) and relative humidity from 79.7% (August) to 93.0% (April).

**FIGURE 2:**
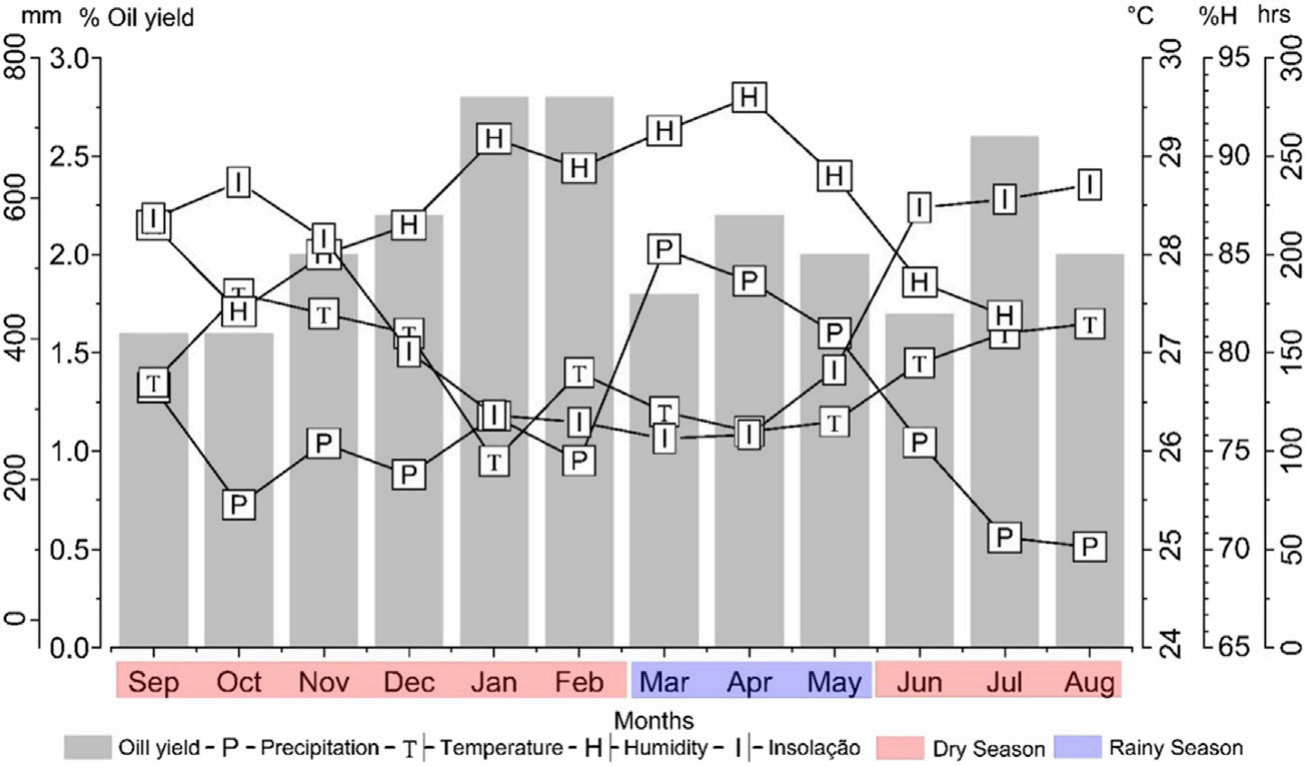
Relationship between climatic parameters and *Hyptis crenata* essential oil yield.

According to precipitation data, the dry period in the region comprised the months of September to February and June to August, with an average precipitation of 215.58 ± 76.26 mm, and the rainy period from March to May, with an average precipitation of 472.53 mm ± 60.22 mm (Figure 2).

**FIGURE 3:**
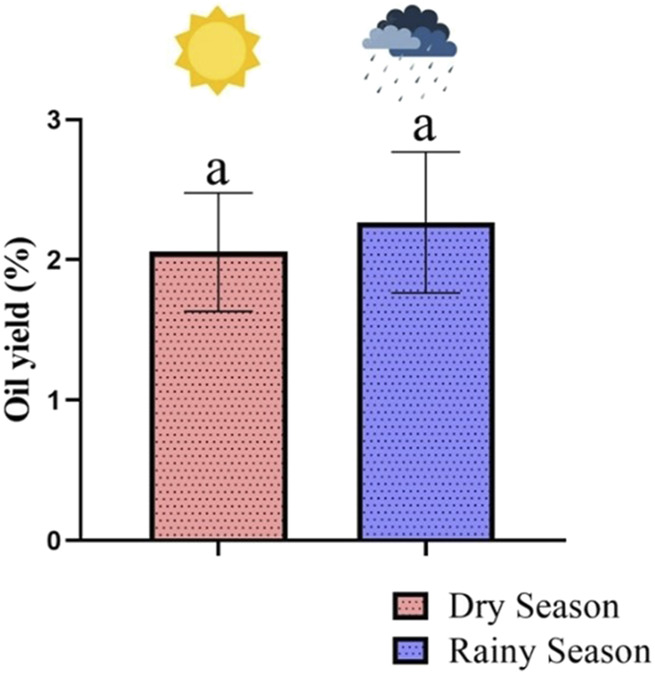
Relationship between seasons and oil production of *Hyptis crenata* during the seasonal study.

In the seasonal study, *H. crenata* leaves essential oil yields varied from 1.6% (September) to 2.8% (January), averaging 2.05% ± 0.39% during the studied year. Statistical Tukey test showed no significant difference in essential oil production during the dry (1.97% ± 0.2%) and rainy (2.07% ± 0.44%) seasons (Figure 3). Furthermore, no significant correlations (*p* > 0.05) were observed between essential oil yield and precipitation (*r* = −0.23), temperature (*r* = −0.12), humidity (*r* = −0.20), and insolation (*r* = −0.36) indicating that *H. crenata* specimen presents the same essential oil production regardless of the climatic conditions. Variations in oil yield may be linked to other abiotic factors, edaphic and/or genetic.

Essential oils (extracted by hydrodistillation) from the dry and fresh aerial part of *H. crenata* collected in Marajó Island (Brazilian Amazon) presented yields between 0.6% and 0.9%, respectively, in samples from Melgaço and São Sebastião da Boa Vista (Zoghbi et al., 2002); in Salvaterra a yield of 1.4% was described (Rebelo et al., 2009). Other reported samples showed yields of 0.2%, 0.6%, and 1.4% in Tocantins (Zoghbi et al., 2002), Cuiabá (Violante et al., 2012), and Mato Grosso do Sul respectively. None of these specimens showed a higher yield than the present study. However, little information has been found regarding phytochemical studies of this species. Moreover, *Hyptis marrubioides* showed high variability in the concentration of the essential oil components due to seasonality variability probably related mainly to the rainfall regime (Botrel et al., 2010a).

### 3.2 Relationship between essential oil composition and climatic parameters

The chromatographic analysis identified 74 constituents in *H. crenata* leaf essential oils, representing an average of 97.96% (92.95%–99.16%) of the total chemical composition of the oils analyzed during the 12 months. The constituents are listed below in ascending order of their respective retention indexes (RI) in Table 1.

**TABLE 1:** Seasonality of essential oils from *Hyptis crenata*.

	RI_C_	RI_L_	Period	Dry					Rainy			Dry			
No.			Collection months	Sep	Oct	Nov	Dec	Jan	Feb	Mar	Apr	May	Jun	Jul	Aug
			Oil yield	1.6	1.6	2.0	2.2	2.8	2.8	1.8	2.2	2.0	1.7	2.6	2.0
			Constituintes	%											
1	927	924^a^	α-thujene	0.18	0.14	0.09	0.15	0.19	0.10	0.12	0.16	0.10			
2	**934**	**932** ^a^	**α-pinene**	**19.34**	**20.98**	**21.69**	**20.01**	**17.14**	**18.80**	**19.73**	**17.93**	**22.17**	**19.43**	**13.55**	**20.20**
3	947	945^a^	α-fenchene	0.70	0.61	0.45	0.39	0.42	0.44	0.49	0.36	0.40	0.42		
4	**949**	**946** ^a^	**camphene**	**3.79**	**3.48**	**2.75**	**3.14**	**3.30**	**3.40**	**3.11**	**2.65**	**2.97**	**2.94**	**3.22**	**2.93**
5	954	955^b^	thuja-2,4(10)**-**diene	0.05	0.06		0.06	0.02			0.01				
6	973	969^a^	Sabinene	0.02	0.02	0.03	0.04	0.07	0.11	0.08	0.07	0.04	0.75	0.43	0.49
7	**978**	**974** ^a^	**β-pinene**	**8.00**	**7.18**	**11.20**	**8.11**	**9.76**	**8.40**	**9.39**	**11.20**	**9.65**	**9.78**	**8.99**	**6.35**
8	991	988^a^	myrcene	1.69	1.58	1.62	1.72	1.94	1.79	1.72	1.43	1.46	1.52	1.69	1.59
9	1,006	1,002^a^	α-phellandrene	0.51	0.44	0.42	0.47	0.46	0.51	0.52	0.38	0.43		0.44	0.46
10	1,012	1,012^a^	δ-3-carene	0.05	0.02		0.01	0.04	0.05		0.04	0.04		0.04	
11	1,017	1,014^a^	α-terpinene	0.65	0.54	0.61	0.67	0.68	0.70	0.70	0.61	0.65		0.61	0.67
12	1,024	1,020^a^	*p*-cymene	2.49	1.77		1.23	1.32			1.07	1.08	1.00	1.35	1.49
13	**1,030**	**1,024** ^a^	**limonene**			**6.28**			**6.75**	**6.60**	**5.32**	**6.30**	**5.95**	**6.45**	**7.14**
14	**1,033**	**1,026** ^a^	**1,8-cineole**	**32.47**	**36.90**	**31.18**	**32.47**	**30.87**	**24.15**	**24.29**	**29.70**	**22.76**	**26.90**	**22.48**	**26.38**
15	1,058	1,054^a^	γ-terpinene	0.84	0.72	1.01	0.96	1.03	1.05	1.05	1.12	1.07	0.87	0.99	0.85
16	1,066	1,065^a^	*cis-*sabinene hydrate				0.06	0.06			0.09	0.03		0.11	
17	1,089	1,086^a^	terpinolene	0.97	0.83	0.86	1.01	1.04	1.03	0.94	0.97	0.93	0.80	1.15	0.86
18	1,099	1,098^a^	*trans*-sabinene hydrate	0.02	0.03		0.07	0.07			0.09	0.03		0.10	
19	1,114	1,114^a^	*endo*-fenchol	0.32	0.26	0.18	0.18	0.24	0.20	0.21	0.21	0.18	0.16	0.24	0.21
20	1,121	1,119^a^	*trans*-pinene hydrate						0.11	0.12	0.16	0.11	0.09		
21	1,122	1,122^a^	*cis-p*-menth-2-en-1-ol	0.11	0.11	0.09	0.13	0.15					—	0.02	0.06
22	1,126	1,127^a^	α-campholenal	0.02	0.02								—		
23	1,140	1,136^a^	*trans-p*-menth-2-en-1ol				0.02	0.04			0.05		—		
24	**1,144**	**1,141** ^a^	**camphor**	**11.11**	**9.39**	**8.86**	**13.56**	**15.22**	**14.34**	**10.12**	**14.79**	**10.76**	**12.84**	**15.90**	**9.30**
25	1,148	1,145^a^	camphene hydrate	0.61	0.63	0.49	0.44	0.45	0.46	0.39	0.41	0.35	0.46	0.46	0.46
26	1,157	1,155^a^	*iso*-borneol	0.06	0.04		0.02	0.04						0.04	
27	1,163	1,163^a^	pinocarvone	0.08	0.08	0.07	0.07	0.09	0.08	0.08	0.09	0.06	0.05	0.09	0.07
28	**1,167**	**1,168** ^a^	**borneol**	**2.57**	**2.36**	**1.74**	**2.11**	**3.03**	**1.74**	**2.46**	**1.98**	**3.38**	**2.43**	**2.31**	**2.07**
29	1,177	1,174^a^	terpinen-4-ol	0.81	0.70	0.73	0.61	0.74	0.73	0.67	0.81	0.62	0.61	0.69	0.63
30	1,175	1,175^a^	*cis-*pinocamphone	0.04	0.02										
31	1185	1,186^b^	*p-*cymen-8-ol	0.09	0.08	0.04	0.03	0.05						0.03	
32	1,191	1,186^a^	α-terpineol	2.66	2.57	2.77	2.26	2.46	2.48	2.24	2.16	1.89	2.38	2.35	2.71
33	1,197	1,194^a^	myrtenol	0.21	0.19	0.17	0.18	0.29	0.18	0.24	0.18	0.12	0.19	0.33	0.18
34	1,208	1,209^a^	*trans*-piperitol	0.02	0.01			0.02							
35	1,220	1,220^a^	*trans-*carveol	0.02											
36	1,244	1,244^a^	carvacryl methyl ether	0.03	0.03									0.03	
37	1,296	1,296^b^	thymol	0.04	0.03						0.04	0.04			
38	1,302	1,302^a^	carvacrol	0.03	0.02										
39	1,352	1,350^a^	α-longipinene	0.95	1.30	0.91	1.12	1.26	0.74	1.11	0.70	0.76	1.08	1.51	1.99
40	1,357	1,357^b^	eugenol	0.34	0.20	0.08	0.03	0.15	0.09		0.04	0.05		0.03	0.11
41	1,374	1,374^a^	isoledene	0.03	0.02		0.03							0.04	
42	1,377	1,374^a^	α-copaene	0.02										0.04	
43	1,411	1,412^a^	α-gurjunene	0.05	0.04		0.06							0.10	
44	1,421	1,417^a^	*E*-caryophyllene	1.92	1.25	1.34	2.43	1.60	1.68	2.32	1.46	1.70	2.24	3.01	3.04
45	1,429	1,430^b^	γ-maaliene	0.07	0.06		0.09	0.07		0.09	0.05	0.07		0.13	0.08
46	1,434	1,434^b^	β-gurjunene	0.02										0.03	
47	1,435	1,436^b^	α-maaliene	0.08	0.07	0.04	0.10	0.08	0.09	0.10	0.06	0.08		0.15	0.10
48	1,441	1,439^a^	aromadendrene	0.70	0.65	0.56	1.00	0.75	0.94	1.03	0.68	0.92	0.84	1.34	1.17
49	1,445	1,445^a^	selin-5,11-diene	0.09	0.07	0.04	0.11	0.09		0.13		0.09	0.07	0.16	0.11
50	1,450	1,449^a^	α-himachalene	0.16	0.18	0.01	0.17	0.18	0.10	0.17	0.08	0.10	0.14	0.22	0.29
51	1,454	1,452^a^	α-humulene	0.10	0.05	0.04	0.11	0.07	0.07	0.11	0.05	0.06	0.07	0.14	0.12
52	1,462	1,464^a^	9-*epi*-*E*-caryophyllene	0.13	0.10	0.06	0.16	0.10	0.14	0.16	0.10	0.14	0.11	0.21	0.17
53	1,473	1,475^a^	γ-gurjunene	0.03	0.01		0.02							0.04	
54	1,479	1,481^a^	γ-himachalene	0.20	0.24	0.14	0.22	0.22	0.12	0.21	0.11	0.13		0.28	0.42
55	1,490	1,490^a^	β-selinene	0.04	0.03		0.04	0.03						0.06	
56	1,496	1,496^a^	viridiflorene	0.25	0.20	0.17	0.37	0.25	0.33	0.38	0.25	0.33	0.28	0.49	0.39
57	1,502	1,500^a^	β-himachalene	0.44	0.49	0.34	0.49	0.55	0.32	0.48	0.30	0.38	0.58	0.67	1.12
58	1,514	1,516^a^	α-dehydro-*ar*-himachalene		0.07			0.06						0.07	
59	1,529	1,530^a^	γ-dehydro-*ar*-himachalene	0.07	0.07		0.05	0.06						0.08	0.07
60	1,543	1,544^a^	α-calacorene	0.06	0.05		0.05	0.05						0.06	0.09
61	1,567	1566^a^	maaliol	0.05	0.03		0.05	0.04						0.12	
62	1,570	1,570^a^	caryophyllenol	0.07	0.01									0.12	
63	1,578	1,577^a^	spathulenol	0.17	0.14	0.10	0.17	0.16	0.21	0.22	0.13	0.17		0.37	0.17
64	1,584	1,585^a^	caryophyllene oxide	0.64	0.48	0.46	0.81	0.62			0.76	0.87	0.91	1.64	
65	1,592	1,592^a^	viridiflorol	0.13	0.25	0.04	0.08	0.06	0.17	0.95	0.09	0.08	0.05	0.06	0.21
66	1,602	1,600^a^	rosifoliol	0.09	0.06		0.10	0.07	0.10	0.12	0.04	0.09	0.09	0.20	0.13
67	1,611	1,611^b^	humulene epoxide II	0.02	0.01									0.05	
68	1,613	1,615^a^	β-himachalene oxide	0.05	0.08	0.02	0.08	0.09		0.09	0.03	0.04	0.09	0.18	0.08
69	1,633	1,627^a^	1-*epi*-cubenol								0.20	0.07		0.16	0.15
70	1,634	1,634^b^	*cis*-cadin-4-en-7-ol	0.07	0.08			0.08							
71	1,637	1,639^a^	caryophylla-4(12),8(13)-dien-5β-ol	0.13	0.07	0.04	0.11	0.06	0.11	0.12	0.04		0.08	0.25	
72	1,652	1,648	himachalol			0.13	0.18	0.20	0.14	0.26	0.09	0.19	1.06	0.36	0.40
73	1,662	1,661^a^	*allo*-himachalol	0.02	0.24	0.21	0.31	0.17		0.42		0.43		0.05	0.68
74	1,666	1,668^a^	14-hydroxy-9-*epi*-*E*-caryophyllene												0.14
	Monoterpene hydrocarbons			39.28	38.37	47.00	37.82	37.22	43.13	44.45	43.32	47.29	43.46	38.91	43.03
	Oxygenated monoterpenoids			51.32	53.47	46.32	52.21	53.82	44.47	40.82	50.76	44.83	46.11	45.18	42.07
	Sesquiterpene hydrocarbons			5.50	5.00	3.64	6.66	5.47	4.53	6.29	3.84	4.76	5.41	8.92	9.26
	Oxygenated sesquiterpenoids			1.44	1.45	1.00	1.89	1.55	0.73	2.09	1.20	1.94	2.28	3.56	1.96
	Others			0.34	0.20	0.08	0.03	0.15	0.09	0.00	0.04	0.05	0.00	0.03	0.11
	Total identified			97.88	98.49	98.04	98.61	98.21	92.95	93.65	99.16	98.87	97.26	96.60	96.43

RI_C_, calculated retention index; RI_L_, literature retention index.

^a^ Adams (2007).

^b^ (Mondello, 2011); Main constituents in bold; Standard deviation was less than 2.0 (*n* = 3).

Oxygenated monoterpenoids (40.82%–53.82%, 46.21% ± 4.49) and monoterpene hydrocarbons (37.22%–47.29%, 43.08% ± 3.5) were the predominant in the essential oil, followed by sesquiterpene hydrocarbons (3.64%–9.26%, 5.44% ± 1.78) and oxygenated sesquiterpenoids (0.73%–3.56%, 1.72% ± 0.73).

**FIGURE 4:**
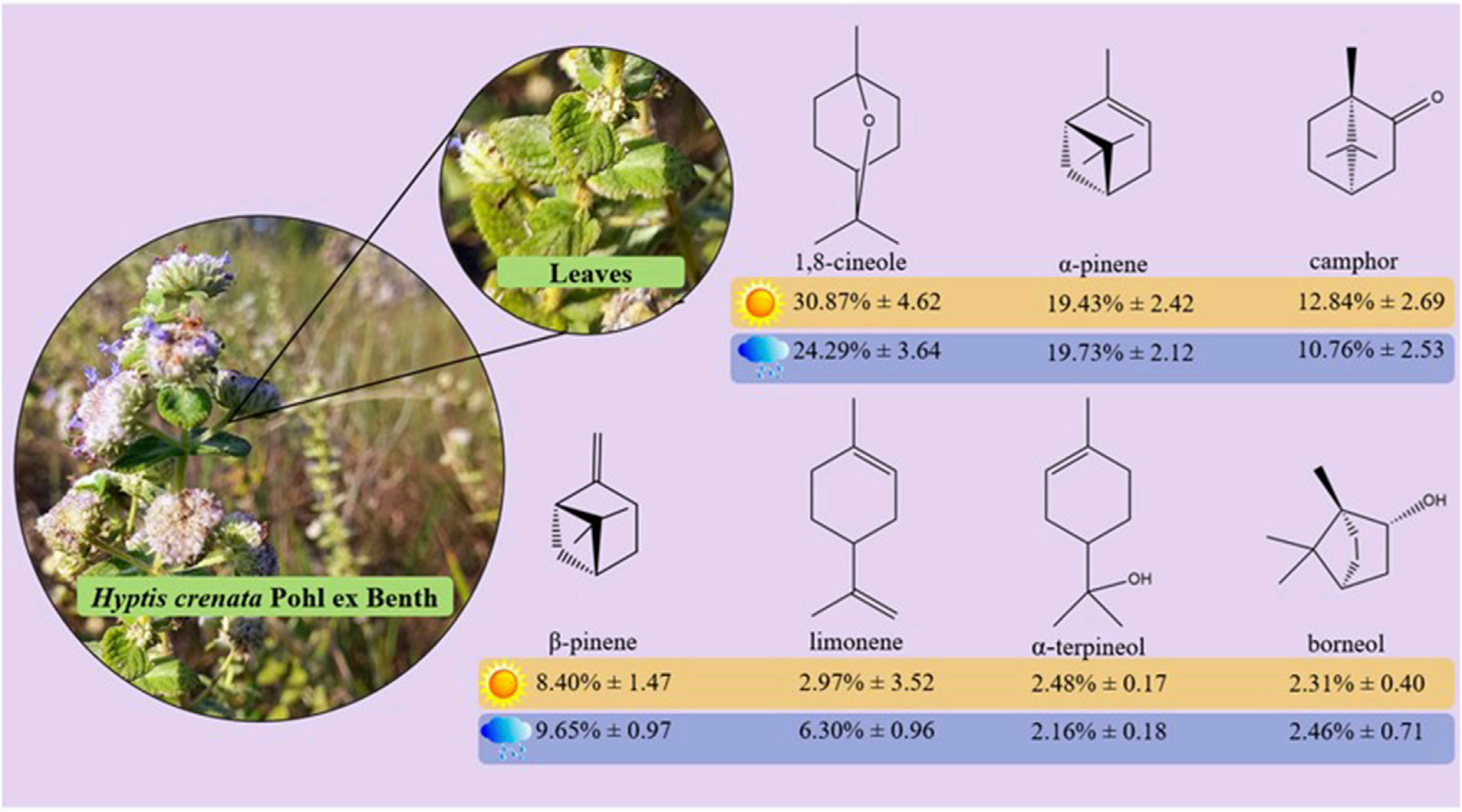
Chemical structures of the main compounds identified in *Hyptis crenata* essential oils leaves during the year.

The oxygenated monoterpenoid 1,8-cineole (eucalyptol) was the main constituent throughout the study, ranging from 22.48% (July/2022) to 36.90% (October/2021), presenting an annual average of 28.48 ± 4. 32%; followed by α-pinene, whose levels varied from 13.55% (July/2022) to 22.17% (May/2022), with an annual average of 19.58% ± 2.29%; the camphor content varied from 8.86% (November/2021) to 15.90% (July/2022), with an annual average of 11.98% ± 2.54%; β-pinene ranged from 6.35% (August/2022) to 11.20% (April/November/2021), with an annual average of 9.19% ± 1.47%; limonene, despite not occurring in the months of September, October, December (2021), and January (2022), presented an average annual content of 6.12% ± 3.15% with variations of 5.32% (April/2022) at 7.14% (August/2022); α-terpineol presented amounts ranging from 1.89% (May/2022) to 2.77% (November/2021) with an annual average of 2.42% ± 0.25% and borneol concentrations ranging from 1.74% (November/2021, February/2022) to 3.38% (May/2022) with an average concentration of 2.34% ± 0.48%. The chemical structures of these compounds are shown in Figure 4.

The chemical composition of *H. crenata* essential oil varies depending on the time of year and the location where the leaves are collected (Santos et al., 2023). Several studies report different chemical characteristics among essential oils of the *Hyptis* genus. A study on *H. marrubioides* showed that the composition of the essential oil varied significantly due to seasonal factors, with no qualitative variation in the composition of the oil throughout the seasons (Botrel et al., 2010b). Furthermore, it was found that the chemical composition of *H. crenata* essential oils presents notable variability, with different main constituents identified in the oils obtained from different samples (Lima et al., 2023).

Intraspecific variability in chemical composition was also noted in other *Hyptis* species, indicating the presence of several chemical compounds. Studies report the existence of different chemical characteristics among the essential oils of *H. crenata*, which generally has the constituents 1,8-cineole, borneol, camphor, limonene, α- and β-pinene, *E*-caryophyllene, *p*-cymene, all of which vary according to the time of year and place of collection (Scramin et al., 2000).

### 3.3 Correlation between climatic parameters and chemical composition

Based on Pearson’s correlation data between the climatic parameters and the *H. crenata* chemical composition, it was possible to identify only a statistically significant correlation (*p* < 0.05) between the amounts of β-pinene and sesquiterpene hydrocarbons with the data of precipitation and humidity, the other constituents such as α-pinene, camphene, limonene, 1,8-cineole, and camphor, as well as the classes of hydrocarbon/oxygenated monoterpenes and oxygenated sesquiterpenes showed statistically insignificant correlations.

**FIGURE 5:**
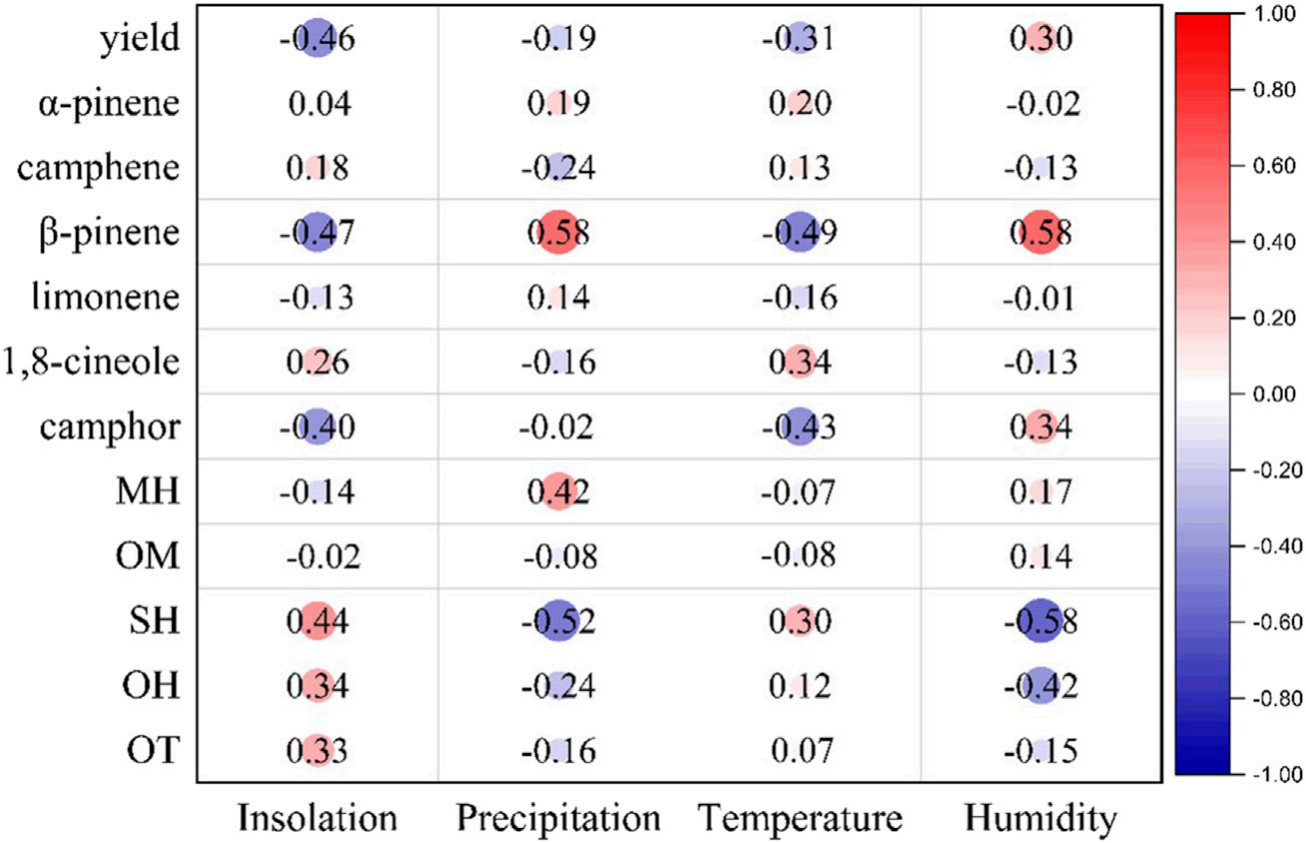
Correlations of *Hyptis crenata* essential oils and climatic parameters monitored during the seasonal study.

β-pinene showed a moderate negative correlation between insolation (*r* = −0.47) and temperature (*r* = −0.49) but without statistical significance (*p* > 0.05); however, this constituent showed a moderate correlation positive correlation with precipitation (*r* = 0.58) and humidity (*r* = 0.58) with statistical significance (*p* < 0.05), in the same way the class of sesquiterpene hydrocarbons showed a moderate and negative correlation (*r* = −0.58) with humidity as displayed in Figure 5.

These data suggest that, despite the four climatic parameters analyzed, the production of the main constituents and classes of terpenoids remains with few qualitative and considerable quantitative variations. However, correlations with β-pinene suggest increasing precipitation and humidity, producing higher monoterpene concentrations in this *H. crenata* specimen. Likewise, humidity influences the class of sesquiterpene hydrocarbons; that is, the increase in this climatic parameter leads to a decrease in the concentration of this terpene class.

α-Pinene and β-pinene are constituents of several essential oils. They have a broad pharmacological action spectrum, described in several studies, with effects such as anti-inflammatory, antitumor and antimicrobial, antibacterial (Leite et al., 2007; Silva et al., 2012), anxiolytic (Santos et al., 2022), antidepressant, anticonvulsant, hypotensive, myorelaxant, antispasmodic (Nikitina et al., 2009; Silva et al., 2012; Felipe et al., 2019; Salehi et al., 2019). β-Pinene, with a woody aroma, occurs in several plants of the Lamiaceae and naturally plays an indirect role in the defense of the plant, attracting natural enemies (predators and/or parasitoids) that help control the population of herbivores (Kutty and Mishra, 2023).

Furthermore, this *H. crenata* specimen displayed low acute toxicity and significant anti-inflammatory activity, with peripheral and no central antinociceptive action (de Lima et al., 2023). However, the chemical composition variation due to seasonality may change the pharmacological activity of *H. crenata* essential oil.

In this way, it is understood that some abiotic or biotic factors that the plant is exposed to favor the significant biosynthesis of α-pinene and, consequently, the decline of camphor since these constituents come from the same formation pathway. In the present study, it was possible to identify that there was no significant correlation between the four climatic parameters analyzed and the α-pinene and camphor amounts.

Furthermore, α-pinene is found in the essential oils of many plants, such as conifers, has a distinct pine aroma, and is known for its various biological properties. In medicinal and/or aromatic plants, α-pinene is involved in various actions, including plant defense mechanisms and repelling insects with its distinct aroma (Kutty and Mishra, 2023).

Furthermore, the chemical composition of essential oils can vary depending on the time and place of harvest. Furthermore, the chemical composition of these oils can undergo biotransformation processes to produce other compounds, such as verbenone, which complements the antibacterial activity of α-pinene (Dewick, 2009).

### 3.4 Multivariate analysis

**FIGURE 6:**
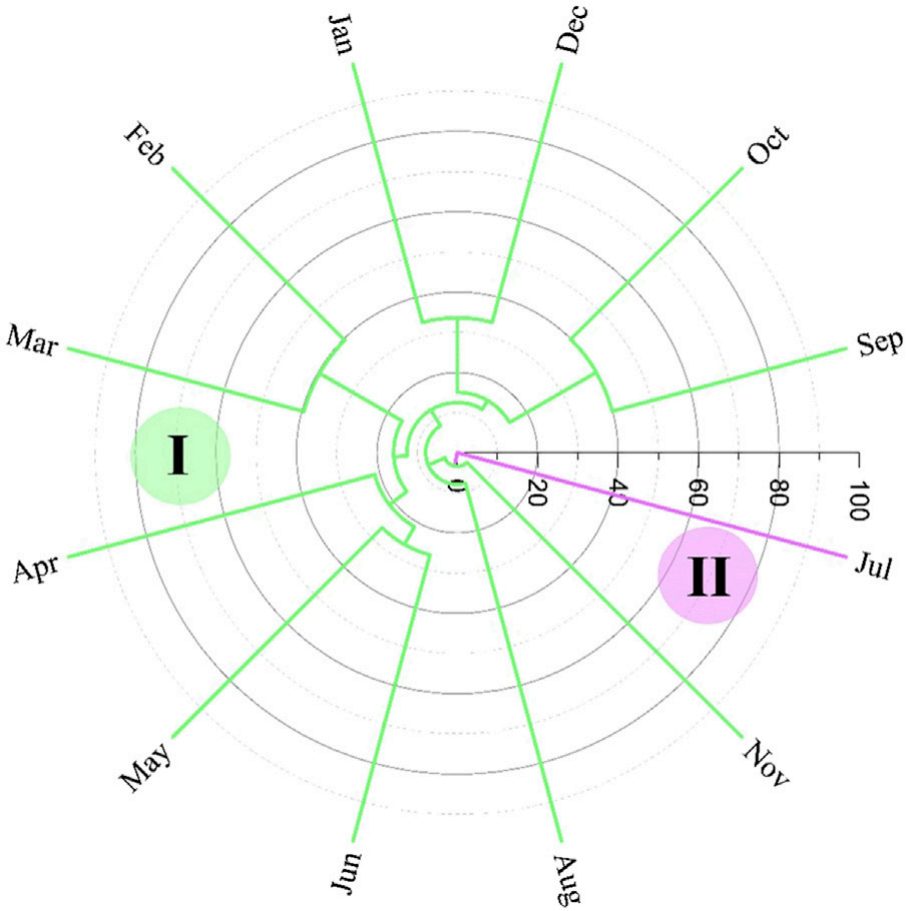
Hierarchical cluster analysis of *Hyptis crenata* essential oils.

Using hierarchical cluster analysis (HCA), a dendrogram was obtained showing two groups formed with the essential oils of *H. crenata* (see Figure 6). Group I comprised all months of study except July (2022), which formed group II.

**FIGURE 7:**
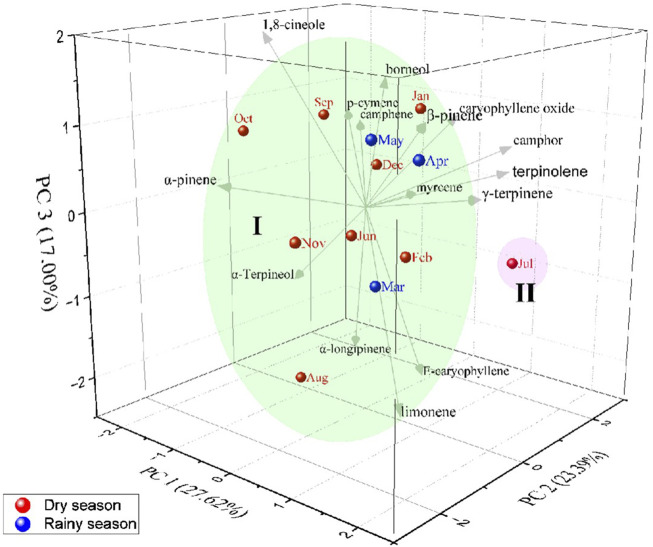
Principal components analysis of *Hyptis crenata* essential oil during the seasonal study.

Principal Component Analysis (PCA, Figure 7) confirmed the formation of two distinct groups and elucidated 68.01% of the data variability. PC1 explained 27.62% of the data and showed negative correlations with α-pinene (*r* = −0.96), camphene (*r* = −1.12), myrcene (*r* = −0.10), *p*-cymene (*r* = −1.25), 1,8-cineole (*r* = −1.49), borneol (*r* = −0.03), α-terpineol (*r* = −1.33), and α-longipinene (*r* = −0.89). The second component (PC2) explained 23.39% of the data and showed negative correlations α-pinene (*r* = −2.01), β-pinene (*r* = −0.92), limonene (*r* = −1.00), 1,8-cineole (*r* = −0.36), and γ-terpinene (r = −0.33). The third component (PC3) explained 17.00% of the data, presenting positive correlations with α-pinene (*r* = 0.39), camphene (*r* = 0.73), β-pinene (*r* = 1.27), *p*-cymene (*r* = 0.88), 1,8-cineole (*r* = 1.94), γ-terpinene (*r* = 0.43), terpinolene (*r* = 0.32), camphor (r = 0.69), borneol (*r* = 1.48), and caryophyllene oxide (*r* = 1.03).

Group I is represented by oils from all months except July, this group was characterized by the chemical type of 1,8-cineole (24.15%–36.90%), α-pinene (17.14%–22.17%), α-terpineol (1.89%–2.77%), α-longipinene (0.70%–1.99%), limonene (5.32%–7.14%), *E*-caryophyllene (1.25%–3.04%), myrcene (1.43%–1.79%), camphene (2.65%–3.79%), caryophyllene oxide (0.46%–0.91%), β-pinene (6.35%–11.20%), and borneol (1.74%–3.38%). However, group II was characterized by the highest camphor levels (15.90%); that is, July was the only month that formed a distinct group, marked by the high occurrence of camphor compared to the other months. Moreover, in July *H. crenata* presented the lowest content of α-pinene (13.55%).

## 4 Conclusion

The *H. crenata* studied specimen showed constancy in essential oil production throughout the year, regardless of seasonal influences, with a chemical profile marked by 1,8-cineole, borneol, α-terpinene, β-pinene, and *E*-caryophyllene. The chemical composition in the dry period was similar to that of the rainy season. There was an occurrence of the same constituents but with a more marked presence of α-pinene and an inevitable decline in camphor.

These results imply that the biological activities presented by the plant and described in previous literature remain constant during the seasonal period due to its unchanged chemical profile. In this way, it is understood that this specimen can be an alternative source of biologically active compounds during different climatic periods in the Amazon. This could have significant implications for obtaining bioactive molecules for the pharmaceutical industry and reaffirms the importance of this medicinal herb for traditional medicine in the Amazon.

## Data Availability

The original contributions presented in the study are included in the article/Supplementary material, further inquiries can be directed to the corresponding author.
